# Longitudinal Effects of Lifetime Caffeine Consumption on Levels of Depression, Anxiety, and Stress: A Comprehensive Review

**DOI:** 10.1007/s13668-025-00616-5

**Published:** 2025-02-01

**Authors:** Sena Unsal, Nevin Sanlier

**Affiliations:** 1 Department of Nutrition and Dietetics, Institute of Health Sciences, Ankara Medipol University, Altındağ, Ankara, 06050 Turkey; 2 Department of Nutrition and Dietetics, School of Health Sciences, Ankara Medipol University, Altındağ, Ankara, 06050 Turkey

**Keywords:** Caffeine Metabolism, Depression, Anxiety, Stress

## Abstract

**Purpose of Review:**

Caffeine has high bioavailability and a purine-like alkaloid structure. It exerts wide-ranging physiological effects by binding to adenosine receptors throughout the human body. Through the activation of those receptors, it can regulate many physiological events in the body. The impact of caffeine consumption on depression, anxiety, stress, and human health remains unclear, constituting an important knowledge gap. This review was conducted to examine the effects of caffeine consumption on depression, anxiety, and stress levels and to offer some recommendations for its future use.

**Recent Findings:**

We performed a comprehensive literature search using PubMed, Web of Science and Google Scholar databases for original articles published in recent years on “caffeine metabolism”, “caffeine mechanism”, “anxiety”, “depression”, “stress”. Caffeine, which has an antagonistic effect on adenosine, can reduce the risk and symptoms of depression and improve general mental health by modulating the central nervous system and neurotransmitter systems. However, increases in anxiety and stress levels, which are often seen together with depression, are observed due to high-dose caffeine consumption.

**Summary:**

Caffeine’s effects on depression, anxiety, and stress may vary depending on different factors, but the level of consumption is particularly important and attention should be paid to upper limits and reference values while evaluating consumption amounts.

## Introduction

Depression is a serious mood disorder that is common worldwide and reported at all sociodemographic levels. Approximately 280 million people in the world are affected by depression, also referred to as depressive disorder [1]. Depression is characterized by the individual’s loss of interest and pleasure and impaired emotional regulation, and it contributes to the global burden of disease by negatively affecting overall quality of life and body functions, with behaviors reflecting a depressive mood [2]. Approximately 3.8% of the general population experiences depression, including 5% of adults and 5.7% of adults aged > 60 years [3]. Depression is approximately twice as likely to be seen in women as it is in men. Furthermore, more than 10% of pregnant and postpartum women worldwide experience depression [4]. Despite the high prevalence of this disorder and the existence of proven treatment methods, fewer than 25% of individuals experiencing depression in low- and middle-income countries receive treatment [5]. With a lifetime prevalence ranging between 1.5% and 19%, depression causes serious social problems with expensive treatments and high mortality and morbidity rates when not properly addressed [6]. One example of this is suicide, which is particularly a major cause of death in the age group of 15–29 years and may be associated with various mental disorders [7]. Another common mental disorder observed worldwide is anxiety [8]. Anxiety is a clinical situation associated with intense stress and characterized by excessive and unrealistic worries, and its severity increases as neurotransmitter synthesis decreases under conditions of chronic stress [9, 10].

Depression, anxiety, and stress occur together in many cases and can be described as risk factors for each other [11]. In the etiology of these three interrelated conditions that affect mental health, environmental factors are especially important. In this context, nutrition is one of the most significant environmental etiological factors. Malnutrition may have a negative effect on the production and regulation of different neurotransmitters that play roles in the regulation of mental health and cognitive functions. While a variety of nutritional deficiencies can lead to depressive disorder, correcting those deficiencies plays a crucial role in preventing depressive disorder [10, 12]. In addition, with the growing recognition that lifestyle choices have a significant impact on mental health, the interaction between nutrition and mental health is becoming increasingly important as well [13]. It is now thought that physical activity, particularly with nutritional models based on dietary diversity, may have positive effects on mental health and that malnutrition plays a role in the pathogenesis of psychological disorders [14].

Although the pathogeneses of disorders such as depression, anxiety, and stress are not yet fully clear, the relationship between the consumption of caffeine from various sources and the risk of depression has received attention [15]. Caffeine is absorbed rapidly and enters the circulation soon after dietary intake, and it acts on the central and peripheral nervous systems via adenosine receptors. While adenosine acts as an inhibitor of the release of neurotransmitters, caffeine has an antagonistic effect on adenosine and it prevents decreases in the release of neurotransmitters while also affecting dopamine activity in the mesolimbic dopamine pathway. Signal transmission in monoaminergic systems modulated in this way is thought to underlie caffeine’s effects on behavior, perception, and thought processes, including various psychotic symptoms [16]. Since caffeine, which is highly popular and can be consumed in various forms, has an extensive range of effects on the human body, its mechanisms of action, dosages of use, and side effects must be carefully evaluated [17]. This review was conducted to examine the effects of caffeine consumption on depression, anxiety, and stress levels and to offer some recommendations for its future use.

## Method

Using the PubMed, Web of Science, and Google Scholar databases, we conducted a comprehensive literature search of original articles published in recent years regarding “caffeine metabolism”, “caffeine mechanism”, “anxiety”, “depression” and “stress” published in recent years. No additional restrictions were imposed other than the selected search terms chosen. First, all search results were scanned by examining the titles and abstracts. Articles written in languages other than English or published as preprint versions were excluded during the screening process. In addition, articles that were not or only slightly related to our areas of interest as indicated by the titles and subtitles were excluded from the scope of the review.

### Physical and Chemical Structure of Caffeine

In addition to being found naturally in plants, caffeine was isolated in its pure form in 1820 and artificially produced through theobromine methylation in 1861 [18, 19]. Caffeine, as one of the most heavily consumed psychoactive substances today, is a part of popular nutritional culture and is consumed in many forms, including energy drinks and nutritional supplements or ergogenic aids [20]. Coffee, the most consumed source of caffeine, is a plant belonging to the genus *Coffea* of the family Rubiaceae, found mostly in North and Central Africa and in some regions of South and Central America and South Africa. More than seven million tons of raw coffee is produced annually worldwide. The seeds from the fruits of these trees are first roasted, ground, and turned into powder. The powder is then mixed with milk or water and a beverage is obtained [21, 22].

**Fig. 1:**
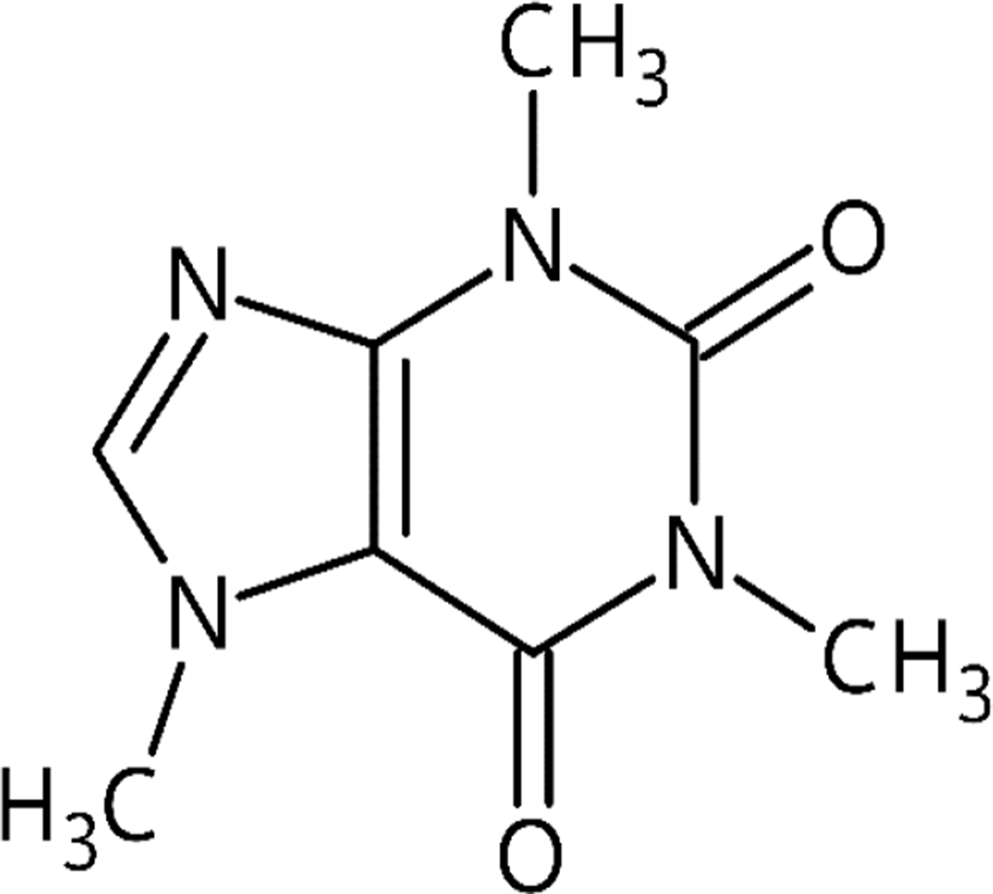
Chemical structure of caffein

**Fig. 2:**
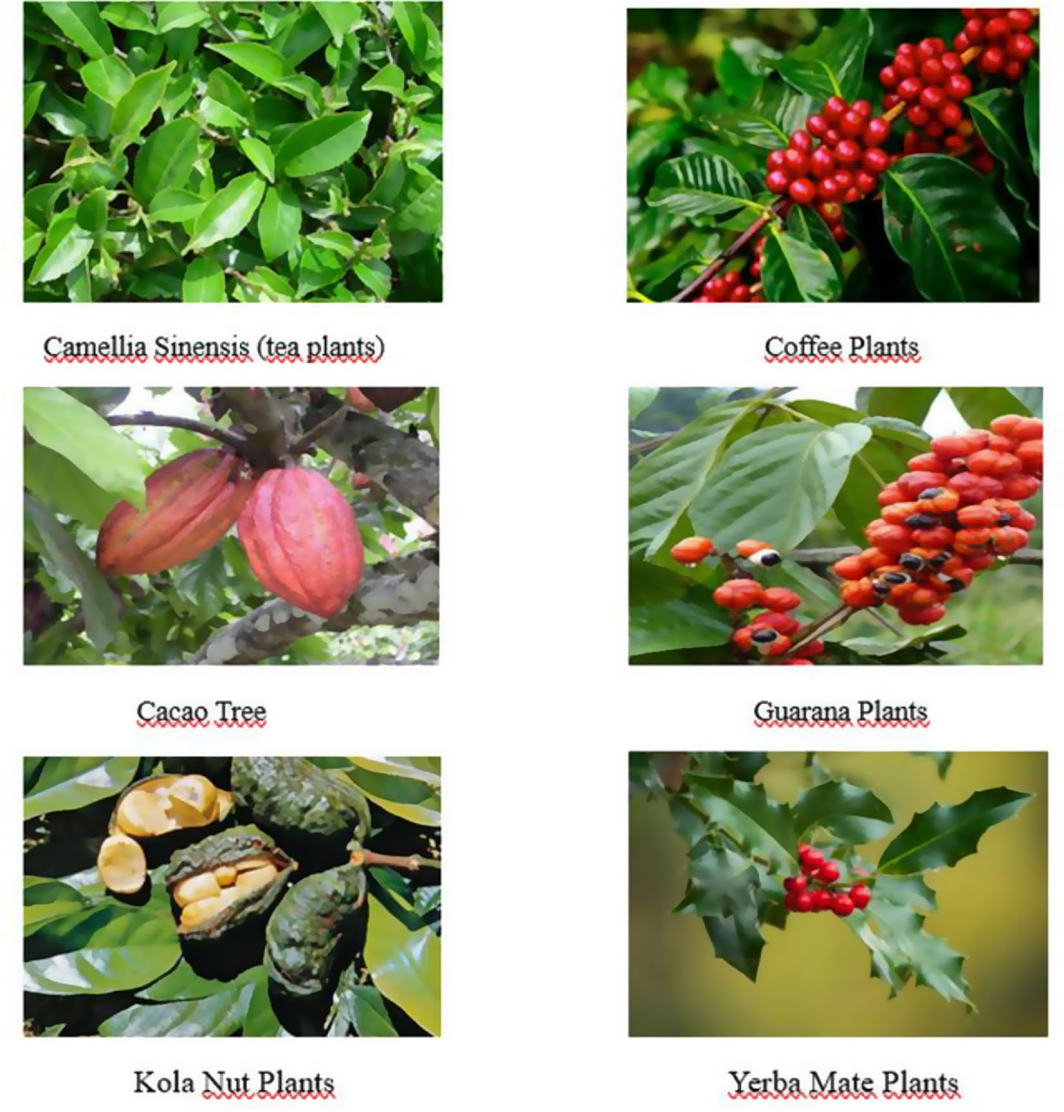
Some plants source of caffeine

The chemical name for caffeine is 1,3,7-trimethylxanthine (C_8_H_10_N_4_O) and it is a psychoactive substance found naturally in plants [21]. As a chemical, caffeine is white in color, odorless, bitter, and weakly basic, with a purine-like alkaloid crystal structure. It is soluble in water and organic solvents. Its chemical name, 1,3,7-trimethylxanthine, signifies that it is a compound formed by the bonding of a methyl group (CH_3_) to the first, third, and seventh carbons in its chemical structure. Caffeine has three metabolites: theophylline, theobromine, and paraxanthine. It can be obtained in pure form by adding CH_3_ to theophylline and theobromine metabolites [19]. As the main source of caffeine, coffee contains more than 1000 chemical compounds. Phenols such as chlorogenic and caffeic acid, lactones, diterpenes such as cafestol and kahweol, niacin and the niacin precursor trigonelline, magnesium, and potassium are some of these compounds. While 38–42% of the dry weight of roasted coffee is carbohydrates, 23% is melanoids, 11–17% is lipids, 10% is proteins, 4.5–4.7% is minerals, 2.7–3.1% is chlorogenic acid, 2.4–2.5% is aliphatic acid, and 1.3–2.4% is caffeine [23]. The chemical structure of caffeine and some plants containing caffeine are given in Figs. 1 and 2.

Another of the most widely consumed sources of caffeine is tea, obtained from the leaves and buds of *Camellia sinensis*, a plant belonging to the family Theaceae. Different types of tea are available according to different preparation methods, including black tea, green tea, white tea, and oolong. The chemical composition of tea includes polyphenolic compounds (catechins and epicatechins), theaflavins, flavonol glycosides, L-theanine, caffeine, theobromine, and volatile organic substances [24]. However, the exact chemical composition and the amount of caffeine tea contains vary depending on the origin of the plant, the region where it was grown, and the processing method. Caffeine levels range between 1.5% and 5% among different types of tea, amounting to 30 mg of caffeine in a cup of green tea or about 50 mg in a cup of black tea [25].

### Caffeine Sources and Consumption

Caffeine is consumed by approximately 80% of the world’s population today [26]. In addition to differences in amounts of caffeine consumption, differences can also be observed in the sources of caffeine consumed around the world [27]. Caffeine is found naturally in more than 60 plants, and the caffeine concentrations contained in its various sources depend on the type of plant, the region in which the plant was grown, and the production process [26]. Caffeine can be found naturally in different amounts in the seeds, leaves, and fruits of many different plants including tea, coffee, kola nuts, cocoa beans, yerba mate, and guarana fruit; it is also present in cola, carbonated drinks, energy drinks, supplements, and some medications [21, 22].

### Caffeine Absorption, Distribution, and Metabolism

Caffeine is a substance with high bioavailability since it has no first-pass effect. However, caffeine metabolism may vary depending on different individual factors such as smoking or alcohol consumption, use of oral contraceptives, pregnancy, menopause, diet, age, and genetics [28]. Depending on the dose taken, the half-life of caffeine is between 2.5 and 10 h. After consumption, caffeine is absorbed in the gastrointestinal tract within 30–45 min, reaching the highest concentrations in plasma and being absorbed in the stomach and small intestine. Its levels rise rapidly in plasma and it is transported through the hepatic vein and spread evenly to all tissues of the body, including the brain. Due to its lipophilic properties, caffeine can pass through biological membranes and the blood-brain barrier with ease [29]. It is metabolized by CYP1A2, the cytochrome P450 isoform found in liver cells, which breaks it down into theobromine (3,7-dimethylxanthine), paraxanthine (1,7-dimethylxanthine), and theophylline (1,3-dimethylxanthine) metabolites [30]. Caffeine is primarily eliminated from the body through renal elimination; while approximately 0.5-2% is excreted in urine without being metabolized, 98% is reabsorbed from the renal tubules after glomerular filtration [31, 32].

### Caffeine’s Functional Properties and Mechanisms of Action

The physiological effects of caffeine on the body are based on a variety of mechanisms, which can be categorized into three main groups: calcium mobilization, phosphodiesterase inhibition, and adenosine receptor antagonism [29]. Caffeine increases the release of intracellular Ca^++^ by the sarcoplasmic reticulum and modulates Ca^++^ channels, thus improving muscle excitation-contraction times and possibly exerting positive effects during endurance exercises [33]. Additionally, caffeine binds to and activates ryanodine receptors, which have an important role in the neurotransmitter system, and affects synaptic transmission, which is dependent on Ca^++^ release [34]. According to the phosphodiesterase inhibition hypothesis, an increase in cyclic adenosine monophosphate (cAMP) concentrations within cells is observed as a result of caffeine’s inhibition of the phosphodiesterase enzyme found in skeletal muscle and adipose tissues. With increasing cAMP, the activation of glycerol, free fatty acids, and lipases also increases and lipolysis is stimulated [34]. Muscle glycogen preservation and muscle functional capacity increase as a result. However, doses much higher than therapeutic doses are needed to produce the effects described here for both calcium mobilization and phosphodiesterase inhibition mechanisms, and it is thought that there is no active mechanism in the case of behavioral effects [35].

The primary effective mechanism of caffeine is considered to be adenosine antagonism [36]. In particular, the effects of non-toxic caffeine concentrations on synaptic transmission and plasticity in the mouse hippocampus are based on adenosine receptor antagonism. Moreover, adenosine A1 receptors appear to be responsible for the effects of caffeine on synaptic transmission, while adenosine A2A receptors regulate the long-term potentiation effects of caffeine [37]. Adenosine is an endogenous nucleoside formed by the combination of a ribose pentose ring and an adenine purine base, and it has inhibitory effects on both the peripheral and central nervous systems [38]. Being structurally similar to adenosine, caffeine competes with adenosine to bind to adenosine receptors and exerts a stimulating effect as an adenosine antagonist. In binding to adenosine, it prevents decreases in the concentrations of neurotransmitter molecules and provides mental and physical benefits by acting on the dopaminergic, glutamatergic, serotonergic, cholinergic, GABAergic (gamma-amino-butyric), and noradrenergic systems [36, 39]. In addition, since quilibrative nucleoside transporter-2 (ENT2) mediated adenosine signalling in V1 is the neurochemical basis of 40 Hz vibration-induced sleep (non-REM and REM sleep), a novel non-invasive treatment is also thought to be useful [40]. Adenosine-A2A receptor signalling has emerged as the neurochemical basis of 40 Hz vibration-induced glymphatic flow through increased cerebrofluid adenosine levels, elimination of increased glymphatic flow by pharmacological or genetic inactivation of compensatory nucleotide transporters-2 or A2AR, and physical and functional A2A receptor-aquaporin-4 interaction in astrocytes. It would provide the potential therapeutic advantage of allosteric modulators of adenosine A2A receptors over classical agonists and antagonists for treating sleep and neurological disorders [41, 42]. The primary functional feature driving the frequent consumption of caffeine is its ability to increase alertness and concentration by affecting the central nervous system [43]. However, it has been suggested that high caffeine intake may negatively affect academic success by causing behavioral and psychosocial problems [44].

**Fig. 3:**
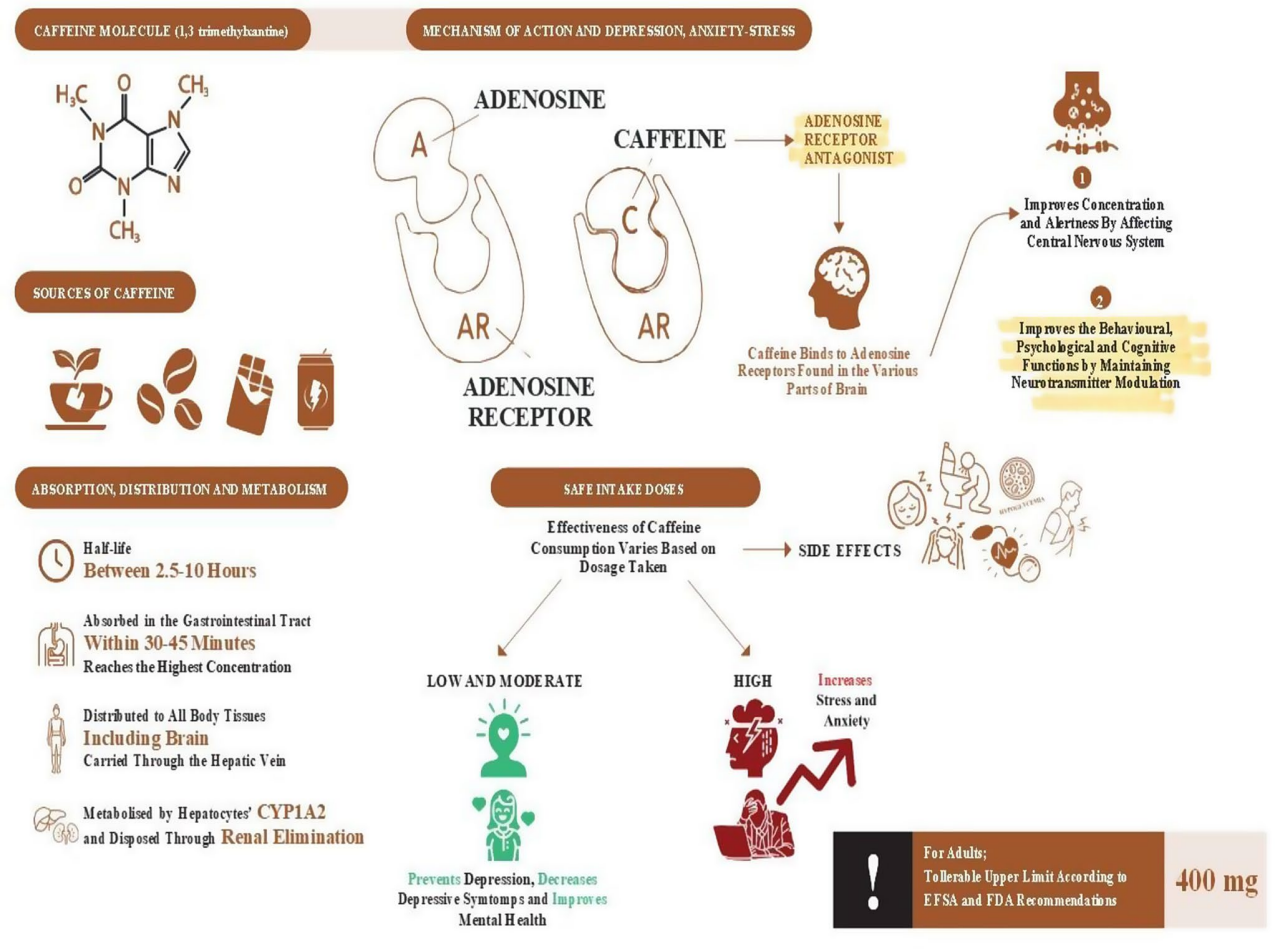
The effects of caffeine absorption, distribution, metabolism, and consumption on depression, anxiety, and stress levels

Another functional feature of caffeine is its ability to exert an anti-inflammatory effect by reducing the levels of proinflammatory molecules. In individuals receiving caffeine supplementation at varying doses, significant reductions in concentrations of cytokines including interleukin (IL)-8, IL-10, IL-6, IL-2, IL-4, interferon gamma (IFN-γ), granulocyte-macrophage colony-stimulating factor (GM-CSF), macrophage inflammatory protein 1β (MIP-1β), monocyte chemoattractant protein-1 (MCP-1), and tumor necrosis factor (TNF) have been reported, even at the lowest dose [45]. It is thought that 3–5 mg/kg caffeine consumption may be effective in preventing the development of neurodegenerative diseases such as Alzheimer’s and Parkinson’s thanks to its neuroprotective effects [46]. Additionally, caffeine imparts various benefits such as alertness and antidiabetic, anti-obesity, and anticancer activities with increased concentrations [47]. The effects of caffeine absorption, distribution, metabolism, and consumption on depression, anxiety, and stress levels are illustrated in Fig. 3. The caffeine contents of various beverages, foods and districts are presented in Table 1.

### Effects of Caffeine Consumption on Depression, Anxiety, and Stress Levels

Depression, anxiety, and stress are common mood disorders that have strong relationships with each other [11].

### Depression

Depression differs clearly from normal mood changes and emotions related to daily life and it seriously affects the global population today [48, 49]. Depression is defined as a serious mood disorder that causes a permanent feeling of sadness, loss of interest and pleasure, and reduced quality of life [50]. Although the etiology of depression has not yet been satisfactorily explained, it is thought that it may develop due to biological factors, chronic diseases, economic situations, stress, grief, abuse or trauma history, drug and substance use, alcohol consumption, and/or genetic factors [48, 51]. In the diagnosis of depression, at least 5 of the criteria specified in the fifth edition of the Diagnostic and Statistical Manual of Mental Disorders (DSM-V) must be observed for a minimum of 2 weeks [9]. There are different theories on the pathogenesis of depression, including disorders of monoaminergic systems, neuroplasticity, increased inflammation, dysregulation of the hypothalamic-pituitary-adrenal (HPA) axis, genetics, and environmental factors. However, the most common treatment approaches address disorders of monoaminergic systems. There are many serotonergic, dopaminergic, and noradrenergic neurons in many regions of the brain. Accordingly, monoaminergic systems are responsible for many behavioral symptoms such as mood, alertness, motivation, fatigue, and psychomotor performance [52]. According to the hypothesis of disorders of monoaminergic systems, depression develops due to decreases in the concentrations of serotonin, norepinephrine, and dopamine monoamine neurotransmitters or abnormalities in their receptors [53].

Caffeine’s effect on depressive symptoms is generally associated with disorders of monoaminergic systems, whereby its mechanism of action is based on adenosine receptor antagonism [54]. The functions of adenosine receptors are distinct. Activation of A2A receptors is associated with increased depressive symptoms, while increased A1 receptor signalling produces antidepressant effects. A2A receptor increase is associated with a decrease in synaptic plasticity and density of synaptic proteins. Furthermore, A2A receptors may cause inhibition of A1 receptors [55]. It is thought that maximum neuroprotection can be achieved with a combination of A2A receptor blockade and increased A1 receptor activation [56].

As explained in more detail in Sect. 5 on the mechanisms of action of caffeine [36, 38, 39], caffeine and adenosine are structurally similar but have antagonistic effects. Adenosine acts as a neurotransmitter inhibitor, while caffeine, which competes with it, exerts the opposite effect, increasing the release of neurotransmitters [54]. On the other hand, decreases in the concentrations of neurotransmitters are observed in cases of depression. Thus, caffeine, which binds to adenosine receptors in various parts of the brain such as the striatum, hippocampus, amygdala, and prefrontal campus, can affect both behavioral and cognitive health in a wide range of areas through the modulation of neurotransmitter systems that are impaired in depression [57]. A significant inverse relationship was reported between caffeine intake and depression scores, and the possible mechanism was attributed to adenosine antagonism [58]. Considering dopamine concentrations, which may particularly decrease in cases of depression, the fact that A2A receptors are expressed in dopamine-rich areas with dopamine receptors and A2A receptors sharing a signaling pathway suggests that caffeine may help in alleviating depression by exerting a dopaminergic effect [54, 57]. Consequently, by increasing the release of neurotransmitters (i.e., dopamine, norepinephrine, and serotonin) with that dopaminergic effect, caffeine stimulates psychomotor activities, increases concentration, and can improve general mood and cognitive functions [59]. According to the European Food Safety Authority (EFSA), in order for such cognitive and psychological effects to occur, caffeine consumption of approximately 75 mg is required, ignoring individual differences [60]. In addition to dopamine, caffeine is also associated with glutamine and GABA. It was reported that the increase in A2A receptors in mice prone to depression caused a decrease in glutamine and GABAergic markers and that those effects disappeared following the administration of caffeine [61]. Another study concluded that caffeine intake increased the sensitivity of dopamine receptors by two- to threefold [62].

Impairment of hippocampal neurogenesis is one of the etiological factors of depression and can be affected by various factors such as stress. Especially in mice exposed to chronic stress, decreased neurogenesis and neuroinflammation were observed [63]. In a study, it was reported that caffeine prevented the decrease in neurogenesis by suppressing corticosterone-induced microglia activation. In addition, the antidepressant effect of caffeine was highlighted in the study [64]. By other hypothesized mechanisms of action, caffeine is thought to affect inflammation and neuroplasticity. In the context of depression, caffeine interacts with A1 and A2A receptors, increases basal transmission, and improves depressive symptoms by preventing the decrease of brain-derived neurotrophic factor (BDNF) [65]. In a study conducted with mice, it was reported that caffeine consumption of 10 mg/kg/day for 2 weeks decreased neuroinflammatory markers, increased BDNF levels, and improved depressive symptoms [66]. In a study conducted with middle-aged individuals, participants who consumed at least 4 cups of coffee per day had a lower risk of depression compared to participants who consumed < 1 cup of coffee per day. Additionally, no significant relationship was found between decaffeinated coffee consumption and depression [67]. In another study on black tea consumption, it was observed that daily consumption of up to 4 cups of black tea was protective against depression [68]. In a study conducted in Korea, green tea, coffee, and caffeine consumption were inversely associated with the risk of depression. That risk was 21% higher in individuals who did not consume green tea compared to those who consumed it regularly (at least 3 cups a week), while individuals who did not drink coffee had a 32% higher risk of depression than individuals who consumed it regularly [69]. In a human study conducted in Portugal, fMRI results were examined in individuals with habitual coffee consumption. According to the results, after coffee intake, posterior default mode network (DMN) connectivity decreased, but connectivity in higher visual and right executive control network (RECN) nodes increased [70]. Another study reported that habitual consumption of coffee and caffeinated products affects the functional connectivity of the human brain. In addition, although it was not associated with depression in the study, the stress level was found to be higher in regular coffee drinkers compared to non-coffee drinkers [71].

The prevalence of elderly depression is increasing as well as in the young adult population [72]. Regular moderate coffee consumption reduces various causes of mortality such as cardiovascular diseases, cancer, depression and cognitive disorders. It is also promising for good mood and well-being in old age [73]. In another study, it was reported that individuals with higher coffee consumption had younger biological ages compared to their chronological age [74]. Therefore, caffeine and especially coffee consumption supports a healthy ageing.

When depression is not treated, serious morbidity and mortality rates are observed. One of the major causes of death in depression is suicide [6, 7]. According to the results of three large American cohort studies, coffee consumption was associated with a lower risk of suicide, while decaffeinated coffee was not associated with a lower risk of suicide. This situation draws attention to the effect of caffeine rather than other components of coffee [75]. On the other hand, a study conducted in Korea reported that excessive consumption of energy drinks was associated with depression and suicidal ideation [76].

Studies have reported varying results regarding the effects of different caffeine sources. In a meta-analysis study, it was emphasized that increase in coffee intake of 240 mL reduced the risk of depression by 4%, while there was no significant relationship between tea consumption and depression. The only significant relationship reported was that among caffeine, coffee, and depression [77]. In a meta-analysis of observational studies, coffee consumption was found to reduce the risk of depression by 8%, but it was stated that more studies on caffeine are needed [15]. Although one study associated increased caffeine metabolites in urine with a non-significant increase in depression risk [78], a study on mice reported that caffeine had an antidepressant-like effect [79].

Recently, the relationship between genes in adenosine receptors and mood disorders has attracted attention. In a study, it was reported that single nucleotide polymorphism in adenosine A2A receptor genes (ADORA2A) was associated with depression in terms of clinical heterogeneity. Especially in individuals with TT genotype, depression was associated with a lower risk of sleep disturbance and attention disorder [80]. In a study conducted in Finland, it was reported that compromised adenosine transport due to SLC29A3 polymorphism may predispose women to depression [81].

Contradictions are also observed in studies exploring the relationship between dose and depressive symptoms. In a comprehensive study, it was found that moderate daily caffeine intake (119.52–236.5 mg) reduced the risk of depression; in particular, individuals consuming an average of 119.52 mg of caffeine constituted the lowest risk group [82]. In a study on the efficacy of low-dose caffeine, a significant relationship was found only between < 90 mg caffeine intake and depressive symptoms. According to that study, no significant relationship existed between > 90 mg caffeine intake and depression scores [65]. On the other hand, a study conducted with a Japanese population showed a significant relationship with depression for caffeine intake up to a maximum of 291 mg/day [83]. In a systematic review, it was reported that the use of caffeine to reduce the negative effects of depression, anxiety, stress, and sleep patterns for academic success and ongoing clinical education has increased significantly in recent years and that moderate doses of caffeine provide benefits, but side effects are also observed at high doses [84]. While low (~ 50–250 mg) and moderate (~ 250–400 mg) caffeine intake is thought to improve physical-cognitive performance, general mood, and depressive symptoms, it is reported that higher levels of caffeine intake have an anxiogenic-like effect and may have negative effects on depression [59, 85]. Correlations regarding caffeine intake amounts, as well as timing and the optimal effects of caffeine intake, remain uncertain. The circadian rhythm may play a role in the psychological and cognitive effects of caffeine. Caffeine can change the circadian rhythm by affecting the gene expression of suprachiasmatic nuclei, which are located in the hypothalamus and control the circadian rhythm [86]. One study showed that caffeine intake in the evening may change the circadian rhythm by delaying the secretion of melatonin, thus reducing sleep quality [87]. In a study examining the relationship between caffeine intake timing and depression in the American adult population, it was found that the incidence of depression was lower among individuals who consumed caffeine in the early morning hours (05:00–08:00) compared to individuals who did not consume it early in the day [88].

### Anxiety

Anxiety is a mood disorder that can be accompanied by physiological somatic symptoms, with feelings of worry and fear occurring at different intensities. It is reported that 68% of individuals experiencing anxiety disorders have another accompanying psychiatric disease [89]. According to the DSM-V, anxiety disorders encompass multiple subtypes, such as generalized anxiety disorder, agoraphobia, obsessive-compulsive disorder, and panic attack disorder [9]. Anxiety disorders, similarly to other mental health problems, are the result of complex interactions of social, psychological, and biological factors. Stressful living conditions, trauma, family history of mood disorders, drug use, and the presence of concomitant psychological disorders such as depression are considered to be risk factors [90]. Today, approximately 4% of the global population suffers from an anxiety disorder. According to data from 2019, 301 million individuals in the world, or about 3.8% of the global human population, have anxiety disorders, making them the most common of all mental disorders [3]. The mechanism of caffeine’s anxiogenic effect is not yet clearly known. However, due to its adenosine receptor antagonism, caffeine can cause symptoms such as hyperventilation and shortness of breath, which are characterized by anxiety, suggesting that caffeine may have common mechanisms for respiratory distress [91]. A2A receptors, in particular, are more associated with anxiety behaviour [55]. On the other hand, it is thought that the serotonergic system is involved in the development and maintenance of anxiety disorders and that caffeine changes serotonergic neuron activity and serotonergic gene expression [92].

In the relationship between caffeine dose and anxiety, it is generally reported that low to moderate levels (40–300 mg) of caffeine have positive effects on mood, while high levels (> 400 mg) of caffeine consumption may have anxiogenic effects [93]. In addition, a study conducted with Australian young adults found that energy drinks with high caffeine contents (limited to 320 g/L) increased anxiety [94]. Another study reported that the consumption of 2–3 cups of coffee per day is associated with a lower risk of depression and anxiety, but intake of > 6 cups of coffee may lead to an increase in anxiety [95]. While a study conducted in Iran reported that those who consumed ≥ 1 cup of coffee per week had a significantly reduced likelihood of depression and anxiety symptoms, no significant relationship was found between the occurrence of symptoms and caffeine/coffee consumption [96]. According to another study, among individuals whose average daily caffeine intake was 268 mg, symptoms such as headache, panic attack, and anxiety were observed, especially with high caffeine intake of > 400 mg [97].

In addition to the daily dose, acute and chronic caffeine consumption should be evaluated. A rat study demonstrated that acute and chronic caffeine treatment in rats under acute or chronic stress reduced oxidative stress markers such as nitric oxide and lipid peroxidation and improved anxiety and cognitive functions [98]. In another study, acute caffeine intervention affected behaviour and mRNA gene expression, but different results were found in behaviour and striatal A2A receptors expression in animals showing high or low anxiety-like behaviour [99]. In a study in which long-term caffeine intervention was examined, A1R expression increased in the frontal cortex, while A2A receptors expression increased in the hippocampus and caudate putamen [100]. In a rat study, acute caffeine administration (20 or 40 mg/kg) decreased aggression behaviours, but chronic caffeine administration (50 or 100 mg/kg) showed no change. This was explained because chronic caffeine administration caused the development of tolerance [101]. It is thought that the contradictory results in acute and chronic caffeine administration may be due to the presence of other signalling pathways against tolerance or acute response [99].

Several factors play roles in the heterogeneity observed in the relationship between caffeine and anxiety. The first of these factors is the caffeine habit. When regular caffeine intake is ceased abruptly, individuals may experience caffeine withdrawal symptoms including increased anxiety and depression [102]. The second important factor is genetics. It is thought that the single-nucleotide polymorphism seen in the CYPA12 gene affects the enzyme activity in caffeine metabolism, and it is hypothesized that variations in the ADORA2A gene stimulate dopaminergic transamination by affecting adenosine receptor-caffeine binding [103, 104]. In a systematic review, it was reported that there is an interaction between anxiety and caffeine and that ADORA2A gene polymorphisms increase the sensitivity of caffeine to anxiogenic effects, thus creating individual patterns in caffeine metabolism [104]. In the systematic review conducted by Fulton et al., individuals with the ADORA2A (rs5751876) TT genotype were found to have higher anxiety levels following caffeine intake and it was further suggested that, even if polymorphism is observed, tolerance to the anxiogenic effect may develop [105]. More studies on this subject are needed for definitive evidence of gene interactions, caffeine addiction, and the relationship between dose and anxiety.

### Stress

Stress is a state of anxiety or mental tension caused by difficult situations; it is a natural human response that allows us to manage challenges and threats in our lives. Everyone experiences stress to some extent, and certain levels of stress can be beneficial throughout our lives. However, high levels of stress can cause physical and mental health problems [106]. The disruption of homeostasis in the body can be caused by many different individual and environmental factors, which are defined as stress factors. Hence, stress constitutes a physical or psychological negative condition that disrupts homeostasis, and stress response is described as the body’s response to a negative situation to sustain homeostasis [107]. Two neuroendocrine physiological systems, the sympathetic-adreno-medullary (SAM) axis and the hypothalamic-pituitary-adrenal (HPA) axis, play roles in maintaining homeostasis against stressors [108]. In the rapid initial response, norepinephrine and epinephrine are increased via the SAM axis. Subsequently, in the slow response, the release of various glucocorticoid hormones increases with HPA axis activation and negative feedback is created [108, 109]. As a result, physical symptoms such as increased heart rate, blood pressure, respiratory rate, lipolysis, gluconeogenesis, and changes in appetite are observed together with mental symptoms such as irritability, difficulty concentrating, and alertness [109].

Adenosine is thought to prevent the behavioral, neurochemical, and electrophysiological changes caused by stress by exerting an antagonistic effect on the A2A receptor, similar to the effects of caffeine on depression and anxiety [110]. In mouse models of repeated stress-induced depression, an abnormal increase in A2A receptor signalling in the lateral septum (LS) was observed and implicated as an upstream regulator of stress-induced depressive-like behaviour. The antidepressant capacity of A2A receptor antagonists is based on this neurophysiological basis [111]. On this basis, a study showed that regular-chronic caffeine consumption improved locomotor activity and reversed stress-induced depression and avoidance of social behaviour [112]. An important point for stress-induced behavioural changes is well-being. In a study conducted with mice, it was reported that moderate doses of caffeinated coffee improved well-being and behaviour in both male and female mice, however, decaffeinated coffee did not cause behavioural improvement in both sexes [113].

One study found that most students consume low levels of caffeine on regular days but consume moderate levels during exam periods. It was reported that 8% of participating students consumed caffeine to cope with stress, while 35.7% consumed caffeine for a better mood and 3.57% to manage depression. The authors concluded that caffeine consumption can be considered a method of stress management and that it tends to increase during stressful periods [114]. While one study showed that high levels of caffeine consumption increased stress levels [115], in another study, no relationship was found between Depression, Anxiety and Stress Scale (DASS-21) scores and daily caffeine consumption [116]. On the other hand, a study conducted in Turkey reported that high levels of caffeine consumption increased anxiety and stress scores and that female gender, smoking, economic status, geographical location, and increasing age were among the factors affecting consumption [117].

To summarize, although there are varying findings on the relationship between caffeine and stress, the main factor determining this effect is the dose, with higher levels of caffeine consumption increasing stress.

### Safe Dosages of Caffeine Intake

The dose consumed is important in the effectiveness of caffeine consumption. Its effectiveness varies depending on the dose, and side effects and even toxicity may be observed at high doses. According to recommendations of the EFSA, the tolerable upper limit of caffeine intake is specified as 400 mg for adults, 3 mg/kg for children and adolescents, and 200 mg/g for pregnant women. In a single dose, the caffeine intake limit is 200 mg/day [60]. Similarly, the U.S. Food and Drug Administration (FDA) states that there are no safety concerns at up to 400 mg, equivalent to about 4 cups of coffee [118]2023). A maximum of 2.5 mg/kg of caffeine per day is the recommended safe intake dose for children and adolescents according to Health Canada recommendations [119]. The average serum caffeine concentration was found to be 5.3 mg/L one hour after daily consumption of 2 cups of strong coffee [120]. If serum caffeine concentrations are > 15 mg/L after consuming about 6 cups of strong coffee, side effects such as insomnia, restlessness, nausea, headache, dizziness, spasm, edema, hypoglycemia, hypokalemia, increased blood pressure, and cardiac arrhythmias begin to occur, while fatal effects can be observed with serum concentrations of > 80 mg/L [121]. It has been found that daily consumption of > 1 g of caffeine can cause toxic effects. Additionally, there is variation in the optimal consumption values presented in the literature and individual factors are believed to have an impact on this situation [20].

### Side Effects of Caffeine

While positive effects of low to moderate amounts of caffeine are observed, high amounts may cause negative effects. The main factor causing side effects due to caffeine intake is the dose consumed. Side effects increase in proportion to the consumed dose [122, 123]. Some examples of side effects include tachycardia and heart palpitations, anxiety, tension, irritability, anxiety, headache, gastrointestinal problems, nausea, increase in blood pressure, insomnia, poor sleep quality, and diuresis, which leads to fluid and electrolyte loss and decrease in plasma [123].

### Conclusion and Recommendations

Caffeine is an adenosine antagonist that functions as an inhibitory neuromodulator. Therefore, it can increase cognitive and mental functions by affecting the central nervous system and neurotransmitter systems. Accordingly, it is thought that consuming moderate amounts of caffeine can prevent stress, anxiety, and depression, reducing depressive symptoms and improving general mental health. On the other hand, high doses of caffeine are thought to be associated with increased anxiety and stress. It is also known that its effects may vary depending on the type of coffee consumed and the brewing method in addition to the amount consumed. The caffeine in coffee affects the emotional state and cognitive functions of those who consume it by stimulating the central nervous system. This effect may be associated with depression and anxiety. Caffeine increases dopamine production by blocking adenosine receptors, which can lead to a more positive mood. However, the results of studies on the correlations between coffee consumption and depression, stress, and anxiety are still uncertain. More clinical and epidemiological studies are needed to clearly explain the mechanisms of caffeine’s effects and the role of genetic factors in the impact of caffeine consumption on depression, stress, and anxiety. Evidence-based data to determine the optimal amount of consumption must be produced.

### Future Perspective

Caffeine is most commonly found in coffee, as well as chocolate, tea, cola, energy drinks, and some medications. Studies report that coffee consumption is advantageous for the majority of people and may have positive associations with conditions such as cancer or cardiovascular, immunological, inflammatory, and neurological diseases. However, high levels of caffeine intake can have harmful effects on the growth and development of children and may cause cognitive impairment in the fetuses of pregnant women who consume it heavily. It may also have negative effects in patient groups with particular conditions and people who are sensitive to caffeine, increasing their susceptibility to diseases in adulthood. Caffeine intake should be restricted or reduced to avoid these negative effects. However, moderate caffeine intake can serve as a healthy way to help prevent various diseases. While the numbers of research studies and meta-analyses continue to grow, the scientific evidence in the current literature remains insufficient to confirm the quality and quantity of caffeine for optimal benefits. To clarify the link between caffeine consumption and specific diseases, reduce side effects, and increase bioavailability, epidemiological and randomized controlled trials are needed together with in vivo, in vitro, animal, and human studies to examine innovative applications of caffeine and consumption patterns in relation to health outcomes. In addition, since most studies conducted to date have focused on adults, little knowledge is available about the effects of consuming caffeinated products among children and adolescents. In the future, the absorption, metabolism, bioactivity, and health benefits of caffeine should be evaluated for different age groups and its mechanisms of action should be further examined.

**Table 1:** Various caffeine sources and their concentrations [38]

Substance	Serving Size (mL/ g/ tablet)	Caffeine Content (mg)
**Beverages**		
Turkish Coffee Filter Coffee Espresso Americano Instant Coffee Decaffeinated Black Tea Green Tea Chocolate Milk Standard Can of Cola Standard Energy Drink	85–90 200 60 355 150 240 220 240 225 355 250	60 90 80 150 60 2 50 45 2–7 40 80
**Foods**	(g)	
Plain Chocolate Milk Chocolate Dark Chocolate (%60–85) Dark Chocolate (%45–59) White Chocolate Chocolate Pudding Cocoa Powder Chocolate Ice-cream	50 50 100 100 100 100 100 50 2 tablet 1 tablet 2–3 tablet	25 10 82 43 0 2 120 2–5 65–130 200 80–200
**Drugs**		
Painkiller Some stimulant medications Slimming drug	2 tablet 1 tablet 2–3 tablet	65–130 200 80–200

## Data Availability

No datasets were generated or analysed during the current study.
